# Supplementary material for: Use of implementation science models, theories, and frameworks in pediatric rehabilitation: Protocol for a scoping review
Source: PLoS One. 2026 May 7;21(5):e0348682. doi: 10.1371/journal.pone.0348682 (PMC13152174; doi:10.1371/journal.pone.0348682)
Supplement: S1 File — Search strategy for OVID-MEDLINE Database. (DOCX) [file pone.0348682.s001.docx]

**S1 File Appendix A: Ovid Search Strategy**

| **Ovid MEDLINE(R) ALL <1946 to July 10, 2025>** | | |
| --- | --- | --- |
| 1 | "Delivery of Health Care"/ or Translational Research, Biomedical/ or "Diffusion of Innovation"/ or Health Plan Implementation/ or Evidence-Based Practice/ or Program Evaluation/ | 238896 |
| 2 | Implementation Science/ | 1778 |
| 3 | (implementation adj2 (science or theory or strategy or framework or process or model* or intervention or research)).ti,kf. or implement*.ab. /freq=3 | 80442 |
| 4 | ("implementation science" or (rehabilitat* adj2 implement*)).ti,ab,kf. | 9003 |
| 5 | ("EPIS" or "Exploration, Preparation, Implementation, and Sustainment implementation science framework").ti,ab,kf. | 767 |
| 6 | ("Consolidated Framework for Implementation Research" or "CFIR" or "CFIR 2.0").ti,ab,kf. | 2748 |
| 7 | ("Theoretical Domains Framework" or "TDF").ti,ab,kf. | 6465 |
| 8 | ("Reach, Effectiveness, Adoption, Implementation, Maintenance" or "RE-AIM " or "Knowledge to Action Framework" or "KTA" or "Practical, Robust Implementation and Sustainability Model" or "PRISM" or "Normalization Process Theory" or "NPT" or "COM-B/Behavior Change Wheel" or "PRECEDE-PROCEED" or "Promoting Action on Research Implementation in Health Services" or "PARIHS").ti,ab,kf. | 20991 |
| 9 | ("NASSS" or "non-adoption, abandonment, scale-up, spread, sustain*").ti,ab,kf. | 130 |
| 10 | (application adj2 (knowledge or research or findings or evidence or science)).ti,ab. | 10490 |
| 11 | (adopt* adj2 (innovation* or evidence or research)).ti,ab. | 4116 |
| 12 | (knowledge adj2 (translat* or disseminat* or implement* or exchang* or application* or transfer* or mobili* or diffus*)).ti,ab. | 20463 |
| 13 | Children with Disabilities/rh or exp Cerebral Palsy/ | 26902 |
| 14 | ((youth or child or p?ediatric) adj2 (medicine or therap* or rehabilitat*)).ti,ab,kf. | 14427 |
| 15 | exp Rehabilitation/ | 381311 |
| 16 | Brain Injuries/ | 58234 |
| 17 | Hemiplegia/ | 12250 |
| 18 | Spinal Dysraphism/ | 6980 |
| 19 | Motor Disorders/ | 1155 |
| 20 | Musculoskeletal Manipulations/ | 2456 |
| 21 | exp autism spectrum disorder/ or autistic disorder/ or exp Child Development Disorders, Pervasive/ or "attention deficit and disruptive behavior disorders"/ or attention deficit disorder with hyperactivity/ or ("ADD" or "ADHD" or (attention adj3 disorder*)).ti,ab. | 202326 |
| 22 | (ASD or Asperger* or Autism or autistic or developmental disab* or disintegrative disorder* or kanner or Pdd or (pervasive adj2 developmental disorder*) or rett?).ti,ab,kf. | 105764 |
| 23 | exp Persons with Disabilities/rh or Intellectual Disability/rh | 15850 |
| 24 | *Amputees/ or *Para-Athletes/ or *"Persons With Hearing Impairments"/ or *"Visually Impaired Persons"/ | 8825 |
| 25 | neuro-rehabilit*.mp. or rehabilit*.ti. or rehabilit*.ab. /freq=3 [mp=title, book title, abstract, original title, name of substance word, subject heading word, floating sub-heading word, keyword heading word, organism supplementary concept word, protocol supplementary concept word, rare disease supplementary concept word, unique identifier, synonyms, population supplementary concept word, anatomy supplementary concept word] | 101303 |
| 26 | (rehabilit* adj2 (treatment or therapy or interven* or p?ediatric*)).ti,ab. | 21298 |
| 27 | exp Rehabilitation Centers/ or exp Rehabilitation Nursing/ or exp Physical Therapy Modalities/ or exp Rehabilitation Research/ or exp Neurological Rehabilitation/ or Physical Therapists/ or Physical Therapy Specialty/ or "Physical and Rehabilitation Medicine"/ | 234655 |
| 28 | "spina bifida".ti,ab,kf. | 9165 |
| 29 | Communication Disorders/ | 3137 |
| 30 | exp Neurodevelopmental Disorders/ | 225675 |
| 31 | Specific Learning Disorder/ | 114 |
| 32 | Speech-Language Pathology/ | 4166 |
| 33 | exp Adolescent/ or exp Child/ or exp Young Adult/ or exp Pediatrics/ or exp infant/ | 4716604 |
| 34 | (child* or adolescen* or "young adult" or minor* or p?ediatrics or newborn* or infant*).ti,ab. | 2837016 |
| 35 | (neonat* or infan* or child* or adolescen* or pediatric* or paediatric*).jw. | 846020 |
| 46 | "learning health system*".tw,kf. | 1356 |
| 47 | Learning Health System/ | 360 |
| 48 | The SHOnet learning health system: Infrastructure for continuous learning in pediatric rehabilitation.m_titl. | 1 |
| 49 | Animals/ not (Animals/ and Human/) | 5321833 |
| 50 | or/1-9 | 334642 |
| 51 | 10 or 11 or 12 | 33002 |
| 52 | 1 and 51 | 3764 |
| 53 | 50 or 52 | 334642 |
| 54 | or/13-32 | 953010 |
| 55 | 33 or 34 or 35 | 5794634 |
| 56 | 53 and 54 and 55 | 5697 |
| 57 | 46 or 47 | 1445 |
| 58 | 2 or 3 or 4 | 82352 |
| 59 | 14 or 15 or 25 or 26 or 27 | 483670 |
| 60 | 57 and 58 | 212 |
| 61 | 57 and 59 | 35 |
| 62 | 60 or 61 | 240 |
| 63 | 56 or 62 | 5934 |
| 64 | limit 63 to yr="2006 -Current" | 4919 |
| 65 | 64 not 49 | 4915 |
| 66 | remove duplicates from 65 | 4908 |
